# The impact of pregnancy and childbirth on pelvic sensation: a prospective cohort study

**DOI:** 10.1038/s41598-023-28323-7

**Published:** 2023-01-27

**Authors:** Charlotte K. Mahoney, Fiona M. Reid, Anthony R. B. Smith, Jenny E. Myers

**Affiliations:** 1 The Warrell Unit, St Mary’s Hospital, Manchester University Hospitals NHS Foundation Trust, Manchester Academic Health Science Centre, Manchester, M13 9WL UK; 2 Institute of Human Development, Faculty of Medical & Human Sciences, University of Manchester, Manchester, Greater Manchester UK

**Keywords:** Medical research, Pathogenesis, Risk factors

## Abstract

Pelvic organ prolapse, urinary, bowel and sexual dysfunction, collectively called pelvic floor dysfunction (PFD) affects 1 in 3 women and has a significant public health impact. The causes of PFD are not fully understood but involve injury to connective tissue and motor nerve during childbirth. Women with PFD also have sensory nerve impairment, and it is likely this occurs during childbirth, but this has never been investigated. In the current study 150 women underwent quantitative sensory testing for vibration sensation at the vagina and clitoris, and stretch sensation at the vagina and introitus, in the third trimester, 3 and 6 months postnatal. Antenatally vibration sensation was reduced but stretch sensation was normal. Postnatally vibration sensation deteriorated whilst stretch sensation initially deteriorated but recovered by 6 months postnatal to antenatal levels (all *p* < 0.001). Mode of birth had a significant impact on sensation, with caesarean section appearing neuroprotective, normal vaginal birth resulted in a transient deterioration in sensation that recovered by 6 months, whilst assisted vaginal delivery was prolonged suggesting persistent neurological impairment (all *p* < 0.015). Further research is required to study the clinical effect of these changes on pelvic floor dysfunction in the medium and long-term.

## Introduction

Pelvic organ prolapse, urinary, bowel and sexual dysfunction, collectively termed pelvic floor dysfunction (PFD) is estimated to affect one in three women in high and middle income countries with a significant public health cost^1,2^.

Women with pelvic floor dysfunction have evidence of pudendal sensory and motor nerve impairment^3–7^. One major factor in the development of pudendal motor nerve injury is childbirth. Research to date has described injury to pudendal motor nerves following vaginal birth whilst pregnancy itself had no effect^8–14^.

The impact of pregnancy and childbirth on pudendal sensory nerved has never been investigated, although it is likely that sensory nerves are also injured alongside motor nerves during childbirth, since they follow the same anatomical pathway through the pelvis.

This is the first study to report the effect of pregnancy and mode of delivery on sensory function of the pudendal nerve.

We hypothesised sensory nerves are unaffected by pregnancy and injured following vaginal birth. Our primary objective was to investigate the effect of mode of delivery on pelvic sensation. Secondary objectives were to evaluate pelvic sensation in the third trimester, and investigate the changes in sensation across the postnatal period.

## Methods

### Study design

One hundred and fifty nulliparous women were recruited from antenatal clinics between 10 and 40 weeks gestation.

Exclusion criteria were incapacity to consent, language barrier, pelvic floor surgery, female genital mutilation, medical conditions predisposing to sensory impairment and neuromodulatory medication. Obstetric exclusion criteria were fetal abnormality, multiple pregnancy and previous second trimester miscarriage over 16 weeks.

Women were assessed in the third trimester (antenatal), at 3 and 6 months postnatal. Appointments were re-booked up to three times to improve retention, women who failed to attend three appointments were excluded from the study.

### Sample size

Data from a pilot study demonstrated a difference in vibration sensation of 10% was of clinical significance to symptoms of PFD^6^. In order to reject the null hypothesis (α = 0.05, β = 80) allowing for a 50% standard deviation in the paired measurements (before and after delivery) a minimum of 29 subjects was required per group to detect a 10% change. Based on an estimated 30% loss to follow up rate, and primiparous caesarean section (CS) rate of 26%, the sample size needed was 147.

### Psychophysical methodology

Women underwent quantitative sensory testing (QST), a neurophysiology technique that tests the minimum stimulus needed to perceive a sensation, called the perception threshold. The equipment used in this study continuously increased the stimulus and incorporated reaction time, called the method of limits. Different stimuli were used to evaluate different nerve fibres, with genital stretch perception used to measure Aα sensory fibres and vibration for Aβ nerve function.

#### Vibration sensation

Vibration was measured using the genito-sensory analyzer from Medoc, Israel. A height adjustable probe was attached to a computer program that linearly increased vibration amplitude (microns) until the woman indicated perception of the sensation using a response button held in her dominant hand, whereby the stimulus stopped and the perception threshold was recorded. The program reset to zero and the procedure repeated six times and the mean calculated. Hand dominance was self-declared by the woman. Prior to testing all women received a standardised verbal explanation of the procedure and a control test on the hand. The testing environment was also standardised where possible.

Vibration perception thresholds were tested at the index finger for the median nerve, 2–3 cm into the vagina for the perineal branch of the pudendal nerve and lightly against the clitoris to assess the clitoral branch.

#### Stretch sensation

Genital stretch perception thresholds were measured using the anorectal manometry catheter from Ardmore healthcare was inserted 4 cm into the vagina and the balloon inflated with 2 cm^3^ of air per second until the woman verbally indicated first awareness of a sensation, at which point inflation was paused and the volume recorded. Inflation was then restarted and continued until the woman indicated perception of a stretching sensation. The balloon was only deflated once both sensations had been recorded. It was then retracted to the level of the hymenal remnant to measure sensation at introitus and the procedure repeated^15^.

### Delivery data

Birth outcome data was collected from the electronic patient maternity record (K2 Athena). The UK national central maternity information system was cross-referenced in the event of missing data points**.**

### Outcome measures

The primary outcome measure was proportional change in sensation following a normal vaginal delivery (NVD), assisted vaginal delivery (AVD) and caesarean section (CS).

Secondary outcome measures were baseline sensation in pregnancy compared to non-pregnant normative data and proportional change in sensation across the postnatal period.

Hyposensitivity was defined a priori as a perception threshold greater than the 95th percentile in the normal population as recommended by the American and European Neurophysiology Societies. represented by a non-pregnant normative data set^16–20^. This data set is the only normative data published on genital sensation in women and remains valid today.

### Statistical analysis

Absolute perception threshold increases with age. To eliminate the effect of age on perception threshold we analysed proportional change in sensation. This was calculated by dividing sensation measurements at 3 or 6 months postnatal by antenatal readings, considered the reference baseline in the absence of pre-conception data.

Proportional change less than 1.0 indicated an improvement in sensation compared to antenatal values; 1.0 indicated no change; and above 1 0 indicated a deterioration in sensation compared to antenatal measurements.

Antenatal data that was non-parametric was transformed to facilitate regression analysis.

Data was non-parametric and analysed using Kruskal Wallis and Dunn’s post-hoc pairwise comparison.

Women who underwent an emergency CS following a failed trial of forceps were included in the assisted vaginal delivery (AVD) rather than CS group, as it was hypothesised the presence of forceps and possible fetal head impact could cause nerve damage even if a vaginal birth was not achieved.

### Research

Ethial approval for this study was obtained from the North West—Greater Manchester West Research Ethics Committee and Health Research Authority UK (GMWest-14/NW/1316). Written informed consent was provided and all research was performed in accordance with relevant guidelines/regulations.

## Results

### Cohort demographics

One hundred and fifty women participated in the study, with a median age of 32 years and body mass index of 24.3 (Table 1).

**Table 1:** Cohort demographics.

**Demographics**		
Age, years	Median (IQR)	32 (28–36)
Ethnicity		
White	n (%)	125 (83.3%)
Asian		9 (6.0%)
Black		8 (5.3%)
Oriental		3 (2.0%)
Mixed		5 (3.3%)
BMI, kg/m^2^	Median (IQR)	24.3 (21.55–28.3)
Reproductive factors		
Gestation, weeks		
At first clinical visit	Median(IQR)	32 (28–35)
At birth		39 (39–40)
Birth weight, g	Median (IQR)	3345 (3090–3668)
Mode of delivery		
NVD	n (%)	59 (39.3%)
Forceps		37 (24.7%)
Ventouse		6 (4.0%)
CS		48 (32.0%)
Type of CS		
No labour	n (%)	
ElCS		25 (52.1%)
EmCS < 4 cm		12 (25.0%)
Laboured		
EmCS 4–7 cm		4 (8.3%)
EmCS 8–10 cm		7 (14.6%)
Indication for CS		
Fetal distress	n (%)	11 (22.9%)
Maternal request		11 (22.9%)
Breech		9 (18.8%)
Failure to progress		5 (10.4%)
Large for dates		3 (6.3%)
Failed forceps		3 (6.3%)
Other		3 (6.3%)
Failed induction		2 (4.2%)
Placenta praevia		1 (2.1%)
Analgesia		
Entanox & diamorphine	n (%)	43 (28.7%)
Epidural		35 (23.3%)
Remifentanyl PCA		17 (11.3%)
Spinal		55 (36.7%)
Perineal trauma		
None	n (%)	20 (19.6%)
First degree		5 (4.9%)
Second degree		22 (21.6%)
Episiotomy		47 (46.1%)
OASI		4 (3.9%)
Labial laceration		4 (3.9%)

*IQR* interquartile range; *NVD* normal vaginal delivery; *CS* caesarean section, *ElCS* elective caesarean section; *EmCS* emergency caesarean section; *OA* occiput anterior; *OP* occiput posterior, *OT* occiput transverse; *PCA* patient controlled analgesia; *OASI* obstetric anal sphincter injury.

### Retention

**Figure 1:**
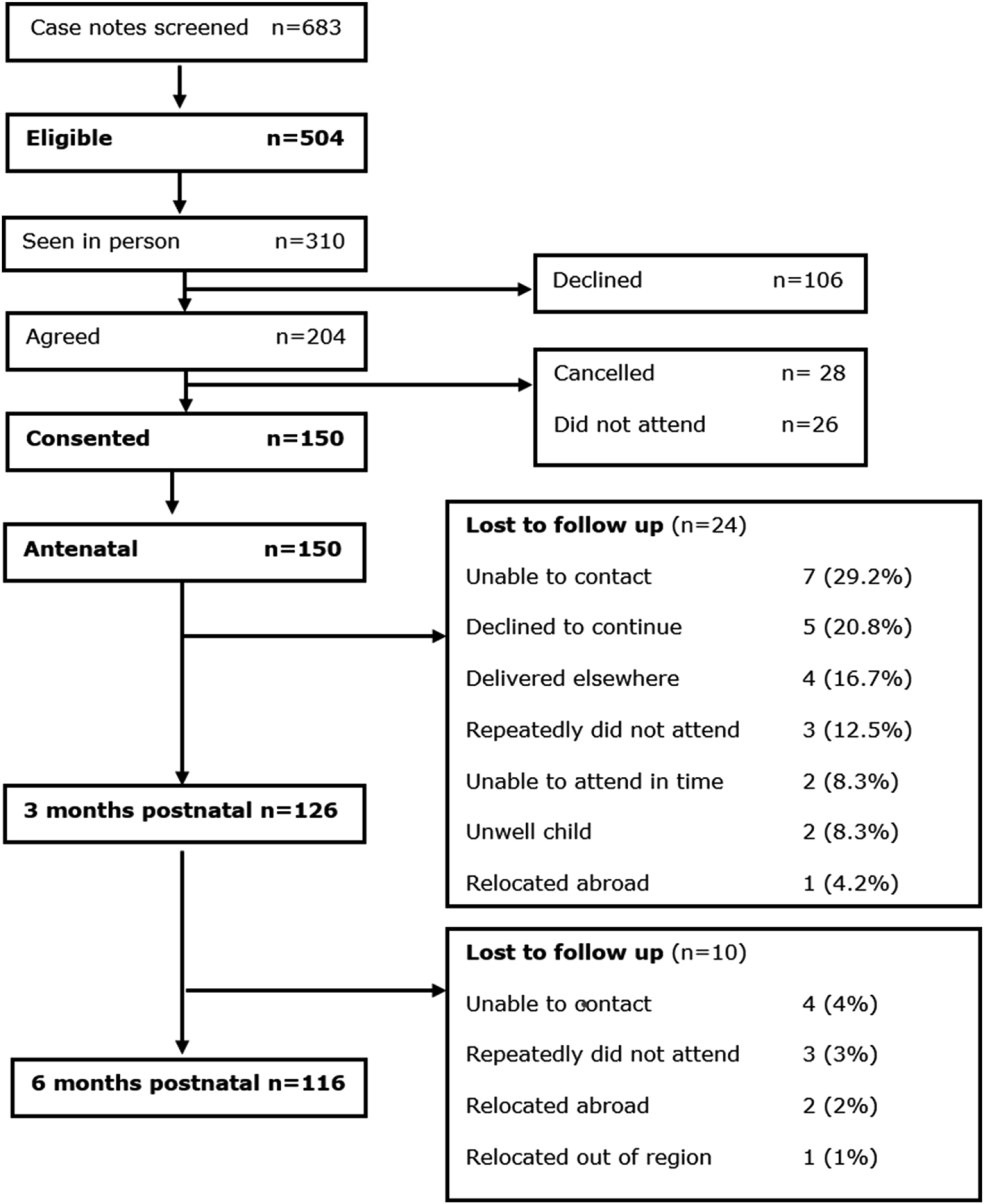
Strobe flow chart. Key: antenatal = anytime in the third trimester of pregnancy; 3 months postnatal = eight to 12 weeks postnatal.

Of the 150 women who attended the antenatal visit, 24 (16%) were lost to follow up at 3 months postnatal (7 CS, 11 NVD and 6 AVD) and an additional 10 (6.7%) were lost to follow up at 6 months postnatal (4 CS, 5 NVD and 1 AVD) (Fig. 1).

### Antenatal sensation

#### Vibration

**Figure 2:**
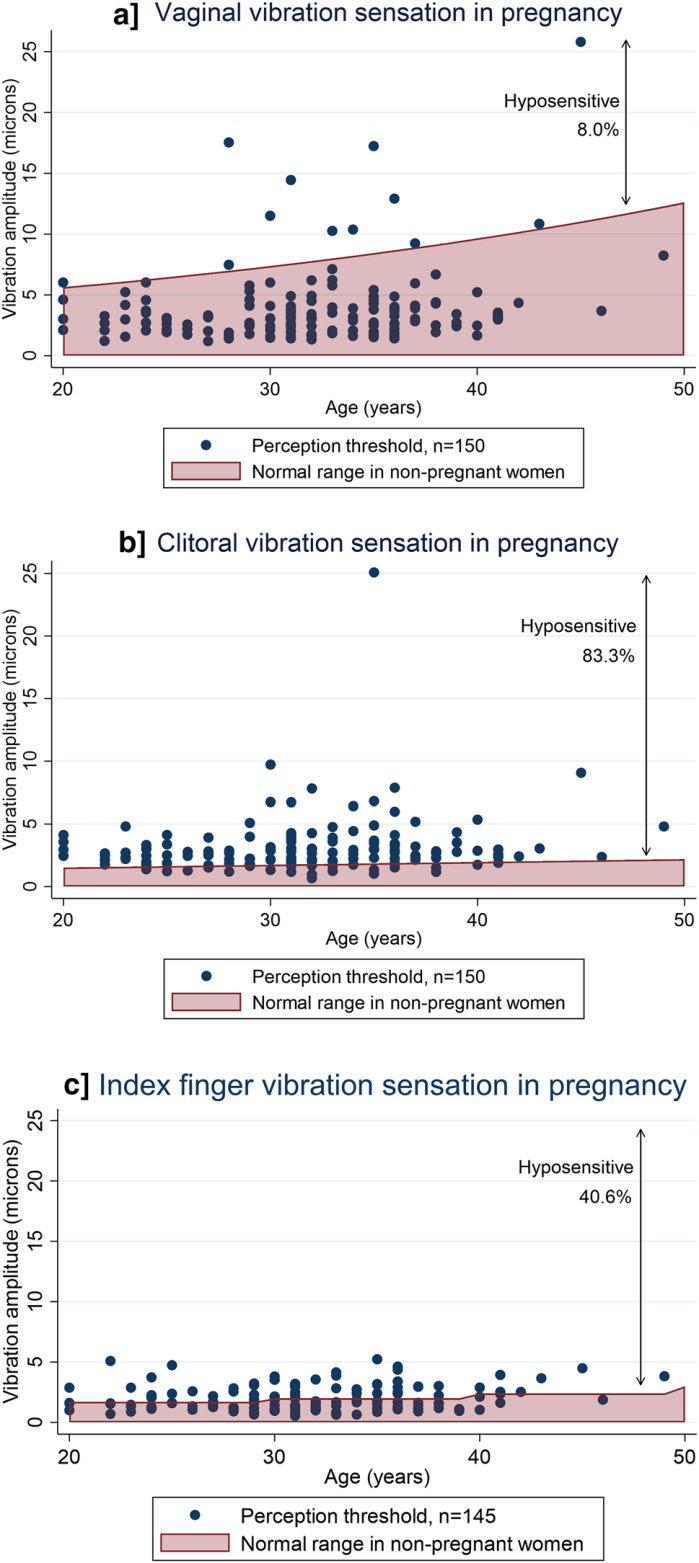
Vibration sensation in pregnancy compared to non-pregnant women. Sensation was compared to previously published age adjusted normative data in non-pregnant women^17^. Higher amplitudes produce a stronger vibration and indicate worse sensation. The clitoris and index finger are more sensitive than the vagina and therefore have lower vibration amplitudes for normal range. Due to a technical error, the computer software did not record perception threshold at the index finger in five women.

Hyposensitivity was present in 8% of pregnant women at the vagina, 83.3% at the clitoris and 40.6% at the index finger compared to non-pregnant age adjusted normative data, Fig. 2^17^.

Gestation at the time of testing and BMI were not associated with change in vaginal, clitoral or index finger vibration sensation.

#### Stretch

**Figure 3:**
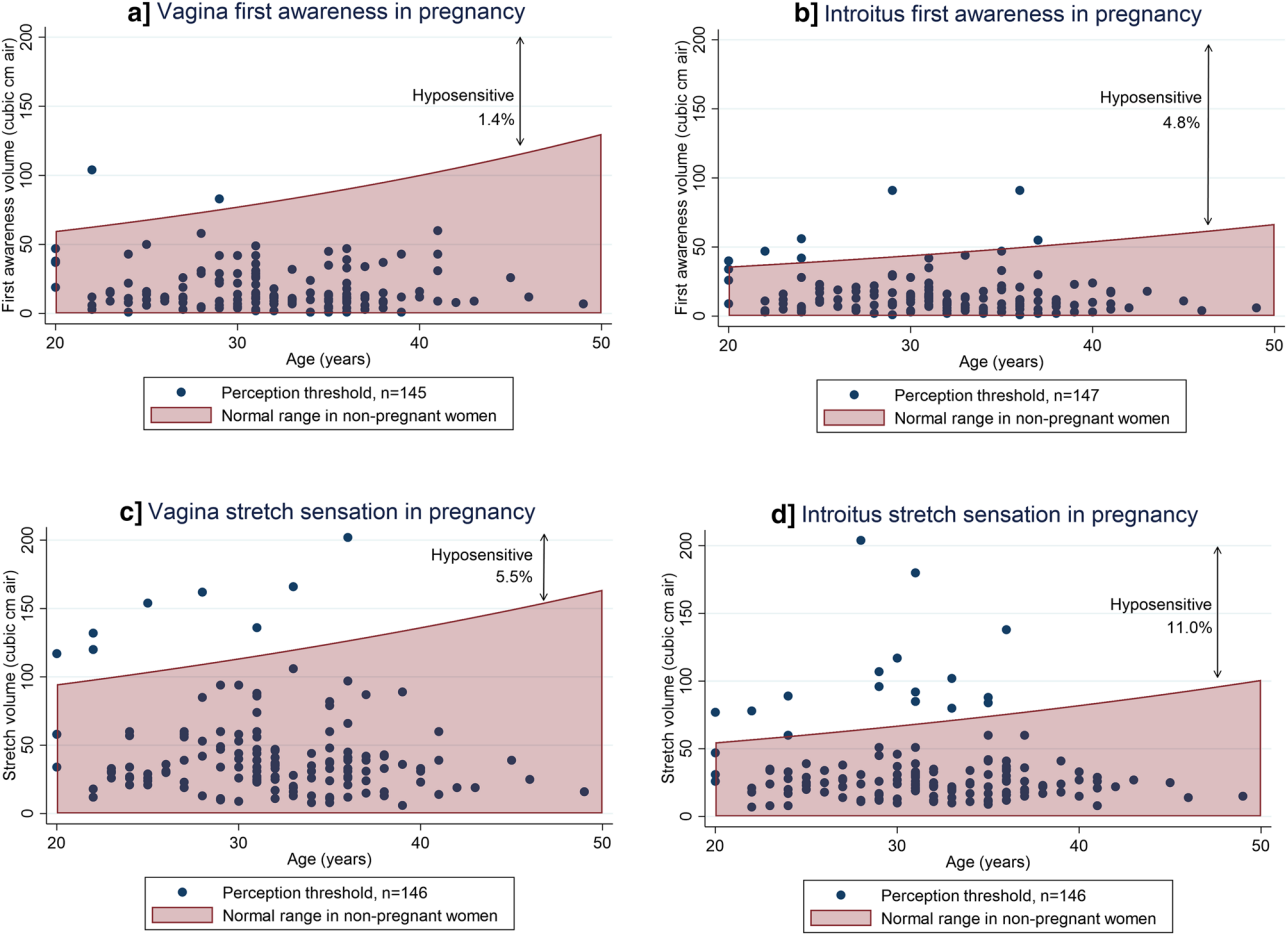
Stretch sensation in pregnancy compared to non-pregnant data. Stretch sensation in pregnancy compared to non-pregnant normative data^15^. Higher volumes produced greater distention and equated to reduced sensation. The introitus is more sensitive than the vagina and therefore has a normal range with lower volumes. Missing data points due to technical error (vagina and introitus stretch = 1) or declined stretch testing (both = 3).

Sensation was reduced in 1.4% of women for vagina first awareness, 4.8% for introitus first awareness, 5.5% of women for vagina stretch and 11.0% for introitus stretch sensation compared to non-pregnant age adjusted normative data, Fig. 3^15^.

Similar to vibration sensation, there was no association between reduced stretch sensation and gestation or BMI.

### Postnatal changes in sensation

Vibration sensation at the vagina improved at both 3 and 6 months postnatal compared to the antenatal baseline (*p* < 0.001, median proportional change at 3 months 0.77, IQR 0.58–0.97; 6 months 0.61, IQR 0.45–0.79).

Clitoral vibration sensation also improved at 3 and 6 months postnatal compared to antenatal (*p* < 0.001, median at 3 months 0.77, IQR 0.57–1.10; 6 months 0.67, IQR 0.47–0.90). The same pattern was seen at the index finger (*p* < 0.001, median at 3 months 0.66, IQR 0.47–0.93; 6 months 0.49, IQR 0.34–0.75).

Vaginal stretch sensation initially deteriorated at 3 months postnatal and recovered to the antenatal baseline by 6 months (*p* = 0.025, median proportional change at 3 months 1.20, IQR 0.77–1.82; median at 6 months 1.13, IQR 0.7–1.60). There was no difference in first awareness at the vagina or introitus, or stretch sensation at the introitus at 3 and 6 months postnatal compared to the antenatal baseline.

There was no association between ethnicity or intrapartum anaesthesia and change in vaginal or clitoral vibration sensation, vaginal first awareness and stretch sensation, or introitus first awareness and stretch sensation.

### Mode of delivery

#### Vibration sensation

**Figure 4:**
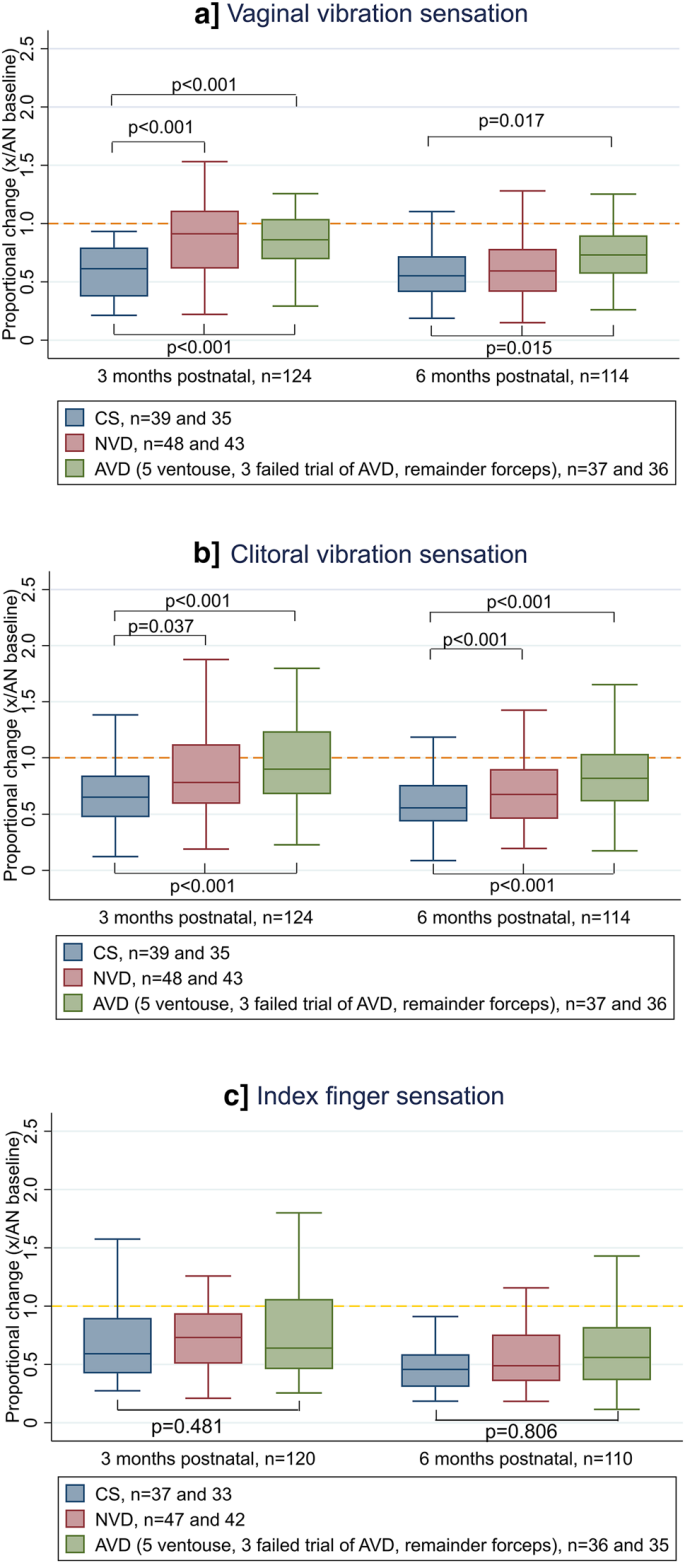
Proportional change in vibration sensation by mode of delivery. Proportional change = 3 or 6 months postnatal perception threshold/ antenatal perception threshold. Yellow line at 1.0 represents no change from antenatal baseline, if value < 1.0 sensation improved compared to antenatal baseline, if value > 1.0 sensation deteriorated. Kruskal Wallis group-wise comparison shown below boxes and significant Dunns post-hoc pairwise comparisons above boxes. Two outliers excluded. Index finger missing data (n = 4) at antenatal with corresponding missing proportional change data. *CS* caesarean section, *NVD* normal vaginal delivery, *AVD* assisted vaginal delivery.

At 3 months postnatal vaginal vibration sensation following a CS improved by 39% compared to the antenatal baseline, whereas following a NVD or AVD there was a smaller improvement of around 10–15% (*p* < 0.001; Median proportional change in CS 0.61, IQR 0.37–0.79; NVD 0.91, IQR 0.61–1.11; AVD 0.86, IQR 0.69–1.04), Fig. 4a.

By 6 months postnatal the improvement seen in the CS group was relatively unchanged at 45%, whilst recovery following a NVD had increased to 41% and was comparable to the CS group, however the same recovery was not evident in the AVD group at 27% (*p* < 0.001; Median proportional change in CS 0.55, IQR 0.41–0.72; NVD 0.59, IQR 0.41–0.78; AVD 0.73, IQR 0.57–0.90), Fig. 4a.

Clitoral vibration sensation at 3 months postnatal improved by 35% following a CS, 18% following a NVD and 10% following an AVD compared to the antenatal baseline (*p* < 0.001; Median proportional change in CS 0.65, IQR 0.47–0.84; NVD 0.78, IQR 0.59–1.12; AVD 0.90, IQR 0.68–1.24), Fig. 4b.

By 6 months postnatal this had increased to 46% improvement overall in the CS group, 33% after a NVD and 18% following an AVD (*p* < 0.001; Median proportional change in CS 0.56, IQR 0.44–0.76; NVD 0.67, IQR 0.46–0.90; AVD 0.82, IQR 0.61–1.03), Fig. 4b.

The index finger, considered the location control, showed no difference in sensation across mode of delivery at 3 or 6 months postnatal, Fig. 4c.

#### Stretch sensation

**Figure 5:**
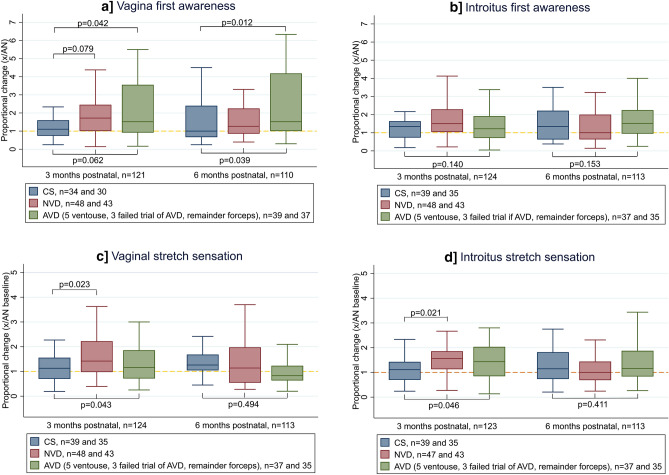
Proportional change in stretch sensation by mode of delivery. Proportional change = 3 or 6 months postnatal perception threshold/ antenatal perception threshold. Yellow line at 1.0 represents no change from antenatal baseline, if value < 1.0 sensation improved compared to antenatal, if value > 1.0 sensation deteriorated. Kruskal Wallis group-wise comparison shown below boxes and significant Dunns post-hoc pairwise comparisons above boxes. Two outliers excluded. Missing data from technical error or woman declined stretch testing with corresponding missing proportional change data (3 months postnatal all n = 1, vagina first awareness n = 2, introitus stretch n = 1; 6 months postnatal all n = 1). *CS* caesarean section, *NVD* normal vaginal delivery, *AVD* assisted vaginal delivery.

Sensation of first awareness at the vagina was unchanged in the CS group compared to the antenatal baseline at 3 months postnatal, whereas sensation deteriorated by 72% following a NVD and 51% following an AVD (*p* = 0.062, Median proportional change in CS 1.10, IQR 0.74–1.60; NVD 1.72, IQR 1.0–2.45; AVD 1.51, IQR 0.92–3.55), Fig. 5a.

By 6 months postnatal sensation of first awareness at the vagina in the NVD group had recovered closer to antenatal levels and was comparable to the CS group. The AVD group showed no signs of recovery with a persistent deterioration of 52%. (*p* = 0.039, Median proportional change in CS 1.00, IQR 0.67–2.40; NVD 1.27, IQR 0.86–2.25; AVD 1.52, IQR 1.0–4.18), Fig. 5a.

Mode of delivery did not affect sensation of first awareness at the introitus at 3 or 6 months postnatal, Fig. 5b.

At 3 months postnatal stretch sensation at the vagina had not changed significantly from antenatal levels following a CS, but deteriorated 44–56% after a NVD and 19–35% following an AVD (*p* = 0.043, Median proportional change in CS 0.99, IQR 0.62–1.45; NVD 1.44, IQR 0.99–1.98; AVD 1.19, IQR 0.75–2.01), Fig. 5c. The same pattern was seen for stretch sensation at the introitus (*p* = 0.046, Median proportional change in CS 1.16, IQR 0.82–1.43; NVD 1.56, IQR 1.13–1.86; AVD 1.35, IQR 0.71–1.98), Fig. 5c. By 6 months postnatal stretch sensation in both the NVD and AVD groups had recovered to CS levels, with no difference between the three groups.

## Discussion

### Principal findings

During pregnancy vibration sensation was impaired in the majority of women whereas stretch sensation was impaired in a few women compared to non-pregnant normative data.

At 3 and 6 months post-natal vibration sensation improved compared to antenatal levels at the vagina and clitoris, whilst stretch sensation initially deteriorated at 3 months postnatal and recovered by 6 months postnatal to antenatal levels.

At 3 months postnatal vaginal and clitoral vibration sensation in women delivered by CS showed greater recovery to antenatal levels than following a NVD or AVD. By 6 months postnatal sensation in the NVD group was comparable to the CS group, but the same recovery was not evident in the AVD group.

First awareness to vaginal stretch did not change significantly across the three visits in the CS group, whilst sensation deteriorated in the NVD and AVD groups at 3 months postnatal. Similar to vibration sensation, by 6 months postnatal the NVD group had recovered to match the CS group, whilst the deterioration in the AVD group persisted.

There was a transient deterioration in stretch sensation at 3 months postnatal after a vaginal birth (NVD and AVD) with no difference at 6 months postnatal across mode of delivery.

### Results in context

#### Sensation in pregnancy

This is the first study to evaluate pelvic sensation during pregnancy and after childbirth; as such there are no studies describing pudendal sensory nerve function for comparison.

Research has demonstrated that pudendal motor nerve function is unaffected by pregnancy, as was stretch sensation in our study^14^. Both stretch sensation and motor function are transmitted along Aα nerve fibres and it follows they would behave similarly in pregnancy.

Vibration sensation is transmitted via smaller Aβ nerve fibres that may be more susceptible to the hormonal changes of pregnancy. The menopause and follicular phase of the menstrual cycle are both associated with reduced sensory nerve function independent of age^21,22^.

Impaired sensation of the median nerve during pregnancy is well documented and ranges from 11 to 66%, thought to be due to compression of the median nerve against bone by the additional extravascular fluid of pregnancy^23–25^.

#### Postnatal changes in sensation

This is the first study to evaluate pudendal sensory nerve function following childbirth. Research describing motor nerve function following childbirth has consistently reported impaired motor nerve function after birth^8–10,14^. As motor function and stretch sensation are both transmitted via Aα nerve fibres this fits with the deterioration in genital stretch sensation seen in this cohort.

The relative improvement seen in vibration sensation after birth could be explained by the hyposensitivity displayed in pregnancy. The apparent increase in Aβ nerve function postnatally may represent a reduction in absolute terms if it was possible to compare with paired pre-conception measurements.

Another explanation for our findings is prolonged compression of the pelvic floor by the fetal head could cause ischaemia of the pudendal nerve branches^26^. Alternatively as the fetal head descends below the ischial spines the pudendal nerve fibres may be stretched leading to a traction injury^27^ It is also possible the nerve damage seen in this study is due to a combination of the above mechanisms.

Our findings suggest there is an element of nerve regeneration or recovery in both Aβ and Aα nerve fibres from 3 to 6 months postnatal. This fits with another study of pudendal motor nerve function following childbirth that described partial reinnervation following vaginal birth in the majority of women^8^.

#### Mode of delivery

In this study elective CS was considered the mode of delivery control, exhibiting the sensory changes seen during pregnancy but not the changes in sensation that occurred with vaginal birth.

Three studies have explored the effect of mode of delivery on pudendal motor nerve function. One described impaired pudendal motor nerve function in women who underwent a vaginal birth compared to a CS at 2 months postnatal, another reported no difference between NVD and AVD at 2 months postnatal and the third found no change in motor nerve function after a CS but a deterioration following a vaginal delivery^8–10^.

Again the findings of these studies correspond with the behaviour of stretch sensation at 3 months postnatal, with no change after CS but a significant deterioration in function following NVD and AVD, further supporting the concept Aα motor and sensory nerves follow the same pattern of injury and recovery.

Interestingly, although vibration sensation via Aβ nerve fibres improved at 3 months postnatal, vibration sensation also displayed greater recovery after a CS than a NVD or AVD.

Our results indicate that women who underwent CS did not experience any deterioration in pudendal nerve sensory function, suggesting CS is neuroprotective for sensory nerves. Women who had a NVD showed slow recovery at 3 months postnatal and demonstrated enough recovery to restore function to CS levels by 6 months postnatal, suggesting nerve damage in this group is transient. AVD was associated with the greatest reduction in sensory nerve function and less recovery of function by 6 months postnatal than a CS or NVD, suggesting AVD causes profound and potentially irreversible nerve damage.

### Clinical implications

Women should be counselled antenatally about the possibility of developing pelvic sensory nerve injury after birth. The majority of AVD in this study were forceps. It is not known whether ventouse produces similar sensory injury to forceps delivery.

### Research implications

The long-term implications of the sensory changes seen in this study remain unclear and further work is needed to investigate for potential associations with the symptoms of PFD.

The majority of AVD in this cohort were forceps and further research also needs to evaluate the impact of ventouse on pelvic sensation, particularly as ventouse has not been associated with an increased risk of developing PFD long-term^28,29^.

### Strengths and limitations

This is the first study to demonstrate feasibility of genital quantitative sensory testing in a pregnant and postnatal cohort and provides a method for clinical evaluation of these women in the future.

Despite the intimate nature of the sensation testing performed and accounting for the significant life change during the course of the study for the women involved, the sample size calculation was exceeded and the attrition rate was only 23%, 7% lower than expected.

One limitation of the study is the subjective nature of all sensory testing. However strict adherence to the QST protocol and control of the testing environment minimised this, but it was not possible to account for the distraction of fetal movements antenatally, or baby distractions postnatally which may have affected response time.

The study did not include an assessment of Aδ and C nerve fibres using cold and thermal QST respectively. This was due to complaints regarding the extended duration of temperature testing from non-pregnant women during a previous study in our unit. This in turn led to ethical concerns subjecting pregnant and post-natal women to this modality^6^.

Another limitation was the use of previously published normative data for evaluation of antenatal sensation, in an ideal world all women would have undergone preconception QST for to provide a paired comparison The population is solely white European and combines data for both nulliparous and parous women^17^. Normative data sets in neurophysiology range from 26 to 106, and thus the sample size of 67 after outlier removal is reasonable^19,30–32^.

## Conclusions

This is the first study to evaluate pelvic sensation in pregnancy and following childbirth with analysis of the impact of mode of delivery. The data suggest Aβ nerve fibre sensation is reduced in pregnancy with sparing of Aα nerve fibres. Vibration sensation improves after birth whilst stretch sensation deteriorates. CS appears to confer some neuroprotection, whilst NVD is associated with a transient nerve injury and AVD more prolonged nerve damage.

## Data Availability

The datasets generated during and/or analysed during the current study are available from the corresponding author on reasonable request.
